# Cervicovaginal Virome Restructuring Associated with HPV Status, Bacterial Dysbiosis, and Cervical Cytological Abnormalities

**DOI:** 10.3390/v18091036

**Published:** 2026-09-19

**Authors:** Almagul Kushugulova, Nazira Kamzayeva, Samat Kozhakhmetov, Elizaveta Vinogradova, Zharkyn Jarmukhanov, Nurislam Mukhanbetzhanov, Artur Kovenskiy, Alibek Kossumov, Aidana Rakhmankulova, Talshyn Ukybassova

**Affiliations:** 1 Laboratory of Microbiome, Center for Life Sciences, National Laboratory Astana, Nazarbayev University, 53 Kabanbay Batyr Ave., Block S1, Astana 010000, Kazakhstan; akushugulova@nu.edu.kz (A.K.); skozhakhmetov.nu@gmail.com (S.K.); st.paulmississippi@gmail.com (E.V.); zharkyn.jarmukhanov@nu.edu.kz (Z.J.); nurislam.mukhanbetzhanov@nu.edu.kz (N.M.); artur.kovenskiy@nu.edu.kz (A.K.); alibek.kossumov@nu.edu.kz (A.K.); 2 Kazakhstan Association of Human Microbiome Researchers, Astana 010000, Kazakhstan; 3 Clinical Academic Department of Women’s Health, Corporate Fund “University Medical Center”, Nazarbayev University, Turan Ave., 32, Astana 010000, Kazakhstan; nasira-kk@mail.ru (N.K.); tallshynu@yandex.ru (T.U.); 4 Interdisciplinary Sports Research, Center for Genetics and Life Sciences, Sirius University of Science and Technology, 1 Olympic Ave., Sirius Federal Territory, Sochi 354340, Russia; 5 Department of Biomedical Sciences, School of Medicine, Nazarbayev University, Kerei-Zhanibek Khandar Street, 5/1, Astana 010000, Kazakhstan

**Keywords:** cervicovaginal virome, phageome, HPV, auxiliary metabolic genes, prophage induction, cervical carcinogenesis, bacteriophage

## Abstract

The cervicovaginal virome remains poorly characterized in relation to human papillomavirus (HPV) infection and bacterial community structure. We performed shotgun metagenomic sequencing of 311 cervicovaginal specimens from a Kazakhstani cohort spanning seven groups defined by HPV status and cervical cytology. Viral community composition differed according to HPV status, with increasing representation of the oncogenic Alphapapillomavirus 9 clade across more abnormal cytological categories. Predicted phage functional profiles also differed between NILM HPV-positive and NILM HPV-negative women. Integrase, excisionase, transcriptional repressor, anti-repressor Ant, and amidase annotations showed differential prevalence after false-discovery-rate correction. Auxiliary metabolic and host-interaction genes associated with nucleotide metabolism, DNA modification, anti-restriction functions, and toxin–antitoxin systems were also differentially represented. Stratification by bacterial community state type revealed contrasting predicted functional repertoires, with toxin–antitoxin-associated annotations enriched in Lactobacillus crispatus-dominated communities and anti-restriction-associated annotations enriched in Gardnerella vaginalis-dominated communities. These findings identify associations between HPV status, bacterial community structure, and cervicovaginal viral composition and predicted phage functions. Longitudinal and experimental studies are required to determine the directionality and biological activity of these associations.

## 1. Introduction

The cervicovaginal niche is a multi-kingdom ecosystem in which bacteria, bacteriophages, eukaryotic viruses, and fungi coexist in dynamic interplay with the mucosal immune system [1]. Over the past decade, shotgun metagenomic and amplicon-based studies have shown that the composition of this ecosystem, particularly the balance between protective *Lactobacillus* species and dysbiosis-associated anaerobes, is associated with susceptibility to sexually transmitted infections and cervical neoplasia [2,3]. Our understanding of this ecosystem is uneven: the bacterial component has been extensively characterized, whereas the virome, the complete viral assemblage of bacteriophages and eukaryotic viruses, has received comparatively little attention.

Cervical cancer remains the fourth most common malignancy in women worldwide, with an estimated 660,000 new cases and 350,000 deaths annually, disproportionately concentrated in low- and middle-income countries [4]. Persistent infection with high-risk human papillomavirus (HPV) genotypes, predominantly HPV16 and HPV18, is an indispensable etiological factor present in virtually all cervical carcinomas, yet on its own it is not sufficient to drive malignant transformation [5,6]. Although over 80% of women of reproductive age acquire HPV at some point, more than 90% of infections clear spontaneously within 6–18 months through immune mechanisms that are still incompletely understood [7,8]. Only a minority of women carry persistent infection long enough for progression to cervical intraepithelial neoplasia and invasive carcinoma. The contrast between the near-ubiquity of HPV exposure and the rarity of malignant transformation points to co-factors that determine whether infection resolves or persists, and this has driven the search for determinants beyond the virus itself.

The cervicovaginal bacterial microbiome has emerged as an important potential co-factor in HPV persistence and cervical disease [9]. The healthy cervicovaginal niche is typically dominated by one of several *Lactobacillus* species, which maintains a low pH (<4.5) via production of lactic acid that is hostile to pathogen colonization [10,11] and produces hydrogen peroxide and bacteriocins, providing competitive advantages against pathogens. Community-state-type (CST) studies have associated *Lactobacillus crispatus*-dominated communities (CST I) with more favorable reproductive and HPV-related outcomes, whereas *Lactobacillus iners*-dominated (CST III) and *Lactobacillus*-depleted communities, including *Gardnerella*-dominated CST IV, have more frequently been associated with HPV persistence and cervical abnormalities [12]. Mechanistically, HPV oncoproteins E6 and E7 suppress innate immune signaling and host defense peptides that vaginal *Lactobacillus* spp. utilize as nutrient sources, thereby promoting *Lactobacillus* depletion and the outgrowth of dysbiosis-associated anaerobes [13,14]. The resulting elevation of vaginal pH and disruption of the mucosal barrier establish a bidirectional relationship between the virus and its bacterial microenvironment in which HPV persistence and bacterial dysbiosis are mutually reinforcing [15].

However, the bacteriome-HPV-centric framework does not fully account for another ecological domain: the virome. The cervicovaginal niche harbors a diverse viral community that extends beyond HPV, including bacteriophages (viruses that infect bacteria), anelloviruses, polyomaviruses, and herpesviruses [16]. Bacteriophages, as obligate parasites of their bacterial hosts, are of particular interest because they directly modulate bacterial community composition through lytic predation and lysogenic integration, yet their role in the cervicovaginal ecosystem has been studied very little. A recent study cataloging 4263 viral operational taxonomic units from the human vagina found that bacteriophages constitute the majority of the vaginal virome, with over 85% of viral species remaining uncultured and functionally uncharacterized [17]. Pilot work has suggested that the cervicovaginal virome clusters by inflammation state and parallels the bacterial microbiome [16]; however, these observations were limited in scope, and the functional repertoire of the cervical phageome and its relationship to HPV-associated progression remain uncharacterized. Despite these observations, no study has systematically characterized the cervical virome across a range of HPV-associated cervical cytological categories or examined how virome restructuring relates to both bacterial community composition and cytological grade.

Because bacteriophages depend on bacterial hosts, changes in cervicovaginal bacterial community composition may be accompanied by corresponding changes in the associated phage community. Such cross-kingdom relationships are well recognized in other mucosal ecosystems [18] but remain incompletely characterized in the cervicovaginal niche. Simultaneous assessment of bacterial and viral communities may therefore provide additional insight into ecological patterns associated with HPV status and cervical cytology.

A further consideration is the pronounced geographic and ethnic underrepresentation in cervicovaginal microbiome and virome research. The vast majority of shotgun metagenomic studies of the cervicovaginal niche have been conducted in North American, European, and East Asian cohorts [19], despite robust evidence that vaginal microbiome composition varies substantially across ethnic groups, with clinically relevant consequences for HPV susceptibility, persistence, and disease progression [20]. Notably, CST IV (*Gardnerella*-dominated) communities are more prevalent among Black, Latina, and certain Asian populations even in the absence of clinical symptoms, yet the functional implications of this variation for virome structure remain unexplored [21]. Central Asia is a particularly significant gap: Kazakhstan bears a high cervical cancer burden (age-standardized incidence of 19 per 100,000 women as of 2022), with cervical cancer ranking as the second most common malignancy in women of reproductive age [4], yet the cervicovaginal microbiome and virome of the Kazakhstani population have never been characterized at the metagenomic level, despite recent evidence from our center that vaginal microecological disorders are significantly associated with HPV infection risk in Kazakhstani women [22]. A concurrent clinical study from our center (*n* = 396) further showed that HPV-16 was the predominant genotype (11.4% overall, followed by HPV-52 at 6.6% and HPV-33 at 5.3%) and the only genotype significantly associated with high-grade colposcopic findings and CIN II/III lesions on biopsy [23]. Situated at the crossroads of European and Asian genetic influences, Kazakhstan provides a useful context for examining whether virome–dysbiosis relationships are generalizable or population-specific.

To address these gaps, we performed extensive shotgun metagenomic characterization of the cervicovaginal virome in 311 women of reproductive age, spanning seven clinical groups from cytologically normal HPV-negative controls through NILM HPV- positive to ASC-H HPV-positive lesions, at the National Research Center for Mother and Child Health, Nazarbayev University (Astana, Kazakhstan), with HPV status verified by a dual-method union criterion (targeted PCR ∪ MNGS). We investigated whether viral taxonomic composition and predicted phage functional repertoires differed according to HPV status, cervical cytology, and bacterial community structure. By integrating virome, bacteriome, and clinical data, we aimed to identify cross-kingdom associations that may generate hypotheses regarding interactions between HPV, bacterial communities, and the cervicovaginal phageome.

## 2. Materials and Methods

### 2.1. Study Design and Participants

A cross-sectional observational study was conducted among women of reproductive age examined at the National Research Center for Mother and Child Health of the University Medical Center Corporate Fund, Nazarbayev University (Astana, Kazakhstan), between November 2024 and February 2025. The study design and reporting follow the STROBE guidelines for cross-sectional studies [24] and the STORMS checklist for human microbiome research [25]; completed checklists are provided in Supplementary Table S1 [24] and Table S2 [25], respectively. A study design flowchart depicting participant enrollment, group allocation, and sample processing is presented in Figure S1.

Inclusion and exclusion criteria have been described in detail previously [23]. Briefly, women aged 18–45 years with a regular menstrual cycle were eligible; those with a history of cervical malignancy, active smoking, HPV vaccination, or antibiotic use within the preceding month were excluded.

Relative abundances for *L. crispatus*, *L. jensenii*, *L. iners*, *L. gasseri*, *G. vaginalis*, and other Gardnerella species were considered, and the species with the greatest abundance was assigned as the sample’s CST.

Participants were stratified into seven clinical groups based on cytological findings according to the 2014 Bethesda System [26] cross-classified with HPV status verified by a union criterion (PCR ∪ MNGS): (1) NILM HPV− (healthy controls); (2) NILM HPV+ (HPV-positive with normal cytology); (3) ASC-US HPV−; (4) ASC-US HPV+; (5) LSIL HPV−; (6) LSIL HPV+; and (7) ASC-H HPV+. The NILM HPV+ group was included to characterize cervicovaginal virome features associated with HPV positivity in women with normal cervical cytology, providing a comparator that is frequently absent from studies focused primarily on cytological abnormalities. The LSIL HPV− group (*n* = 20) was retained as a separate stratum to enable comparison of viral community features between HPV-negative and HPV-positive women within the LSIL cytological category. This design choice enables comparison of virome architecture between HPV-negative and HPV-positive women at each cytological grade.

### 2.2. Ethics Approval and Consent to Participate

The study was conducted in accordance with the principles of the Declaration of Helsinki and was approved by the Local Bioethics Committee of the University Medical Center (Protocol No. 5, dated 23 May 2024). All participants provided written informed consent prior to enrollment.

### 2.3. Clinical Examination and Sample Collection

Cervical specimens were collected during a standard gynecological examination with extended colposcopy, performed jointly by an obstetrician-gynecologist and a colposcopy specialist, using sterile Dacron-tipped cervical swabs. Cytological diagnosis was rendered according to the 2014 Bethesda System [26] by an experienced cytopathologist; histological verification by colposcopy-directed biopsy was performed for 68 participants in the LSIL HPV+ (*n* = 59) and ASC-H HPV+ (*n* = 9) groups. Specimens were placed in sterile 1.5 mL polypropylene microcentrifuge tubes and stored at −80 °C until nucleic acid extraction. All samples were processed within a single freeze–thaw cycle.

### 2.4. HPV Genotyping and Dual-Method Verification

HPV status was verified by two independent methods using a union criterion (PCR ∪ MNGS). Targeted PCR genotyping was performed using the RealBest DNA HPV High Carcinogenic Risk Genotype kit (Vector-Best, Novosibirsk, Russia; cat. no. C8899), which detects HPV genotypes 16, 18, 31, 33, 35, 39, 45, 51, 52, 56, 58, and 59 by real-time PCR. A sample was classified as HPV-positive by PCR if the cycle threshold (Ct) was <35 for at least one genotype, in accordance with the manufacturer’s specifications.

In parallel, HPV sequences were identified from shotgun metagenomic sequencing data during taxonomic classification of reads. Because MNGS covers a substantially wider genotype spectrum than the targeted PCR panel, the union criterion assigns HPV-positive status if either method yields a positive result (PCR+ and/or MNGS+), thereby maximizing sensitivity. Consistency between two detection methods is provided in the Results section (“Concordance between PCR and MNGS methods of HPV status identification”). Because PCR and MNGS have complementary strengths, final HPV-positive status was assigned when either method was positive. This union criterion was selected to reduce false-negative classification due to the limited genotype coverage of the targeted PCR panel or insufficient MNGS sensitivity at low viral abundance.

### 2.5. DNA Extraction and Shotgun Metagenomic Sequencing

Total DNA was extracted from cervical specimens using the ZymoBIOMICS DNA Miniprep Kit (Zymo Research, Irvine, CA, USA) according to the manufacturer’s protocol. Nuclease-free sterile water was used as a negative extraction control; the ZymoBIOMICS Microbial Community Standard (Zymo Research Corporation, Irvine, CA, USA; cat. no. D6300) served as a positive extraction control. DNA quality and concentration were assessed fluorometrically (Qubit 2.0/4.0 (Thermo Fisher Scientific, Waltham, MA, USA)).

### 2.6. Sequencing Quality Control and Contamination Assessment

Performance of the positive control (ZymoBIOMICS Microbial Community Standard) was evaluated by comparing the observed relative abundances of the eight bacterial and two fungal species at the species level against the expected theoretical composition. Recovery rates (observed/expected ratio) were calculated for each species; a recovery rate of 0.5–2.0 was considered acceptable [27]. Negative extraction controls (nuclease-free water processed through the identical extraction protocol) were not subjected to sequencing; consequently, computational decontamination using prevalence-based methods (e.g., decontam) could not be applied. The absence of sequenced negative controls is acknowledged as a limitation. To reduce spurious viral assignments, downstream virome analyses were restricted to contigs ≥ 2 kb classified as viral by geNomad, assessed with CheckV, and carrying viral hallmark genes.

Sequencing was performed on the Illumina NovaSeq 6000 platform (2 × 150 bp paired-end reads) at Haplox GeneTech (Hong Kong, China). Raw reads were quality-filtered using fastp v0.23.4 [28] with default parameters (adapter trimming, low-quality base removal). A total of 1,446,450,777 reads were obtained for 319 samples; after quality control, the analytical cohort comprised 311 samples. The median sequencing depth was 45,580,000 reads per sample (range: 39,620,000–71,370,000).

### 2.7. Bioinformatic Pipeline

Host DNA depletion was performed by aligning reads to the human reference genome (GRCh38) using Bowtie2 v2.5.4 [29]; mapped reads were excluded from subsequent analysis. All processing steps were executed on a high-performance computing cluster.

We used Kraken2 v2.1.3 for metagenomic classification against the standard PlusPF database (k2_pluspf, build 20251015), downloaded from the Kraken2 index repository [30]. This database incorporates complete reference genomes from NCBI RefSeq spanning bacteria, archaea, viruses, plasmids, protozoa, and fungi, as well as the human reference genome (GRCh38) and UniVec_Core.

De novo contig assembly was performed using MEGAHIT v1.2.9 [31] with parameters: --k-list 27,37,47,57,67,77,87,97,107,117,127; contigs shorter than 200 bp were excluded. For downstream virome analyses (functional annotation, lysogeny assessment, and auxiliary metabolic gene identification), only contigs ≥ 2000 bp were retained to ensure sufficient gene content for reliable annotation. Viral sequences were identified using geNomad v1.12.0 [32]; completeness and quality of viral genomes were assessed by CheckV v1.0.3 [33]. Annotation of protein-coding genes and identification of auxiliary metabolic genes (AMGs) were performed using pharokka v1.9.1 [34]. Lysogeny prediction was performed using geNomad’s marker-based classification: contigs were assigned as temperate (lysogenic) if they carried integrase or site-specific recombinase genes, and as virulent (lytic) otherwise. AMGs were identified by pharokka using the PHROGs (Prokaryotic Virus Remote Homologous Groups). Only viral contigs carrying viral hallmark genes were retained for all downstream functional analyses, including AMG identification and lysogeny assessment.

### 2.8. Statistical Analysis

Data analyses were performed in Python v3.9.16 and R v4.2.2.

Clinical and demographic characteristics, as well as comparisons of the top abundant taxa, were conducted using the Kruskal–Wallis test, Mann–Whitney U test, χ^2^ test, or Fisher’s exact test. The significance threshold was set at *p* < 0.05, and all tests were two-sided.

Relative abundances (compositional proportions) were used for alpha- and beta-diversity analyses. Alpha diversity was assessed using the Faith index (scikit-bio v0.63 [35]). Beta-diversity grouping was assessed by principal coordinate analysis (PCoA) based on Bray–Curtis dissimilarities of Hellinger-transformed data. The significance of inter-group separation was tested using ANOSIM (999 permutations), implemented in scikit-bio.

Differential prevalence of phage-encoded functional genes between clinical groups was assessed using Fisher’s exact test with Benjamini–Hochberg control of the false discovery rate, additionally reporting 95% confidence intervals for odds ratios (OR), following the differential-abundance analysis approach applied in our previous shotgun metagenomic study [36]. This analysis was performed using SciPy v1.17.0 [37] and statsmodels v0.14.6 [38]. Features were considered differentially distributed at *q* < 0.05.

Additionally, to explore multivariable effects, we performed adjusted analyses for all PHROG categories, controlling for age, parity, marital status, BMI, HPV status, cervical cell status, and CST. For cell status, NILM served as the reference category; for CST, *L. crispatus* was the reference. These analyses were conducted using MaAsLin3 v1.2.0 [https://www.nature.com/articles/s41592-025-02923-9 (accessed on 4 September 2026)], employing only the prevalence modeling mode (evaluate_only = “prevalence”). Multiple testing correction was performed using the FDR BH procedure, and associations were considered significant at *q* < 0.05.

Data visualizations were done using Matplotlib v3.10.8, seaborn v0.13.2 and d3blocks v1.6.2 [39,40,41].

## 3. Results

### 3.1. Study Cohort and Sequencing Output

A total of 311 women of reproductive age who underwent examination at the University Medical Center, Nazarbayev University (Astana, Kazakhstan), between November 2024 and February 2025 were included in the analytical cohort. Participants were classified into seven groups according to HPV status and cervical cytology: NILM HPV− (*n* = 103), NILM HPV+ (*n* = 42), ASC-US HPV− (*n* = 39), ASC-US HPV+ (*n* = 36), LSIL HPV− (*n* = 20), LSIL HPV+ (*n* = 61), and ASC-H HPV+ (*n* = 10). Detailed clinical and demographic characteristics are presented in Table 1.

The NILM HPV+ group comprised women with normal cytology and HPV detected by PCR and/or MNGS, providing an HPV-positive comparator without cytological abnormality. Age and body mass index did not differ significantly among the seven groups (Table 1). Marital status and number of childbirths differed across groups (*p* < 0.01 and *p* < 0.05, respectively), whereas the proportion with a history of pregnancy ranged from 67% to 90%.

### 3.2. Concordance Between PCR and MNGS Methods of HPV Status Identification

HPV status was determined by two independent methods, targeted PCR genotyping and shotgun metagenomic sequencing (MNGS), and the final HPV status was assigned using a union criterion (PCR ∪ MNGS). Overall concordance between the two methods was 79.1% (Table 2). Positive percent agreement was 74.3%, negative percent agreement was 81.8%, and Cohen’s κ was approximately 0.55, indicating moderate agreement between the two detection approaches. Discordance in the PCR+/MNGS− category was observed in 29 samples (25.7%) and is attributable to viral loads below the MNGS detection threshold: when positive PCR results with small viral load were excluded at thresholds of 1 × 10^5^, 2 × 10^5^, and 5 × 10^5^ copies, concordance increased to 83.9% (87 patients PCR+ MNGS+), 87.3% (79 patients), and 91.7% (48 patients), respectively. Conversely, discordance in the PCR−/MNGS+ category was observed in 36 samples (18.2%), largely reflecting the detection by MNGS of low-risk HPV genotypes (e.g., HPV32, HPV42) absent from the targeted PCR panel (Table 1). Total HPV viral load measured by PCR (×10^5^) and estimated from MNGS-derived relative abundance were significantly correlated (Spearman *r* = 0.33, *p* < 0.01).

### 3.3. HPV Genotype Distribution

By targeted PCR, the most prevalent genotypes were HPV16 (detected in 23% of all HPV-positive cases), HPV52 (15%), HPV33 (12%), HPV45 (9%), HPV59 (9%), HPV39 (8%), HPV18 (8%), HPV51 (7%), HPV56 (7%), HPV31 (6%), HPV35 (6%), and HPV58 (6%). Co-infection with ≥2 genotypes was documented in 42 patients. The proportion of detected co-infections differed across cytological categories—3% in NILM, 5% in ASC-US, 33% in LSIL, and 40% in ASC-H (*p* < 0.001, χ^2^ test)—consistent with prior reports associating multi-genotype infection with increased risk of lesion progression [42,43]. Simultaneously, the proportion of infection with HPV16 increased significantly with the degree of cytological abnormality: 3%, 8%, 24%, and 40%, respectively (*p* < 0.001, χ^2^ test). The clinical significance of this HPV16 narrowing is independently corroborated by biopsy-confirmed data from our center, where HPV16 prevalence rose from 16.7% in CIN I to 80.0% in CIN III and 75.0% in carcinoma in situ [23].

By MNGS, the prevalence of *Alphapapillomavirus* genotypes was the following: HPV16 (29.0%), HPV18 (11.0%), HPV53 (11.0%), HPV45 (11.0%), HPV26 (10.0%), HPV33 (10.0%), HPV32 (9.0%), HPV31 (8.0%), HPV52 (7.0%), HPV6b (7.0%), HPV35 (7.0%), HPV11 (7.0%), HPV90 (7.0%), HPV30 (7.0%), HPV54 (6.0%), HPV7 (5.0%), HPV34 (4.0%), HPV71 (4.0%), and HPV10 (1.0%). *Gammapapillomavirus* were encountered rarely in particular the following genotypes were observed: HPV144 (2.0%), HPV156 (1.0%), HPV127 (1.0%), HPV101 (1.0%), HPV166 (1.0%), HPV121 (1.0%), HPV134 (1.0%), and HPV103 (1.0%). Co-infection with ≥2 genotypes was documented in 47 patients. Similarly to PCR, the proportion of detected co-infections increased with histological severity: 3% in NILM, 8% in ASC-US, 23% in LSIL, and 40% in ASC-H (*p* < 0.001, χ^2^ test). At the species-group level, the *Alphapapillomavirus* 9 (α-9) clade predominated (59.7%), followed by α-7 (21.0%), α-3 (17.6%), α-6 (17.6%), α-10 (12.6%), and α-5 (12.6%).

### 3.4. Community State Types

*Lactobacillus* dominance was observed in 62% of all participants. The relative abundance of *Lactobacillus* was significantly higher in HPV− women (35.29% (IQR: 4.76; 84.93)) than in HPV+ women (13.96% (0.16; 80.85); *p* < 0.05). The most frequent community state type (CST) was CST III (*Lactobacillus iners*-dominated; *n* = 86, 28%), followed by CST I (*Lactobacillus crispatus*-dominated; *n* = 60, 22%), CST IV B (1) (*Gardnerella* sp.-dominated (excluding *G. vaginalis*); *n* = 63, 20%), CST IV B (2) (*Gardnerella vaginalis.* dominated; *n* = 55, 18%), CST II (*Lactobacillus gasseri*-dominated; *n* = 28, 9%), and CST V (*Lactobacillus jensenii*-dominated; *n* = 10, 3%). Notably, CST IV B (1) and CST IV B (2) collectively accounted for 38% of cases.

### 3.5. Sequencing Parameters and Virome Signal Characteristics

Sequencing was performed for 319 samples; after quality control, the analytical cohort comprised 311 samples. A total of 1,446,450,777 reads were generated, of which 736,752 (0.051%) were classified as viral. The median proportion of viral reads per sample was 0.014% (mean 0.045%; range 0.002–2.015%), consistent with expected virome signal levels in shotgun metagenomic profiling of mucosal specimens without viral enrichment [16,44]. The bacteriophage component dominated the virome: *Andhravirus andhra* (458,029 reads; 57.5% of all viral reads) and *Valbvirus* ValB1MD2 (111,671 reads; 15.0%) collectively accounted for 72.5% of virome reads. *Alphapapillomavirus* 9 (α-9 HPV group), the only eukaryotic virus among the top five taxa, contributed 15,927 reads (3.0%) and was detected by MNGS in 71 of 311 samples (22.8%).

The classification of HPV genotypes into species-level α-groups follows the NCBI Taxonomy (Supplementary Table S3 [45]).

### 3.6. Cervical Virome Composition by HPV Status

**Figure 1:**
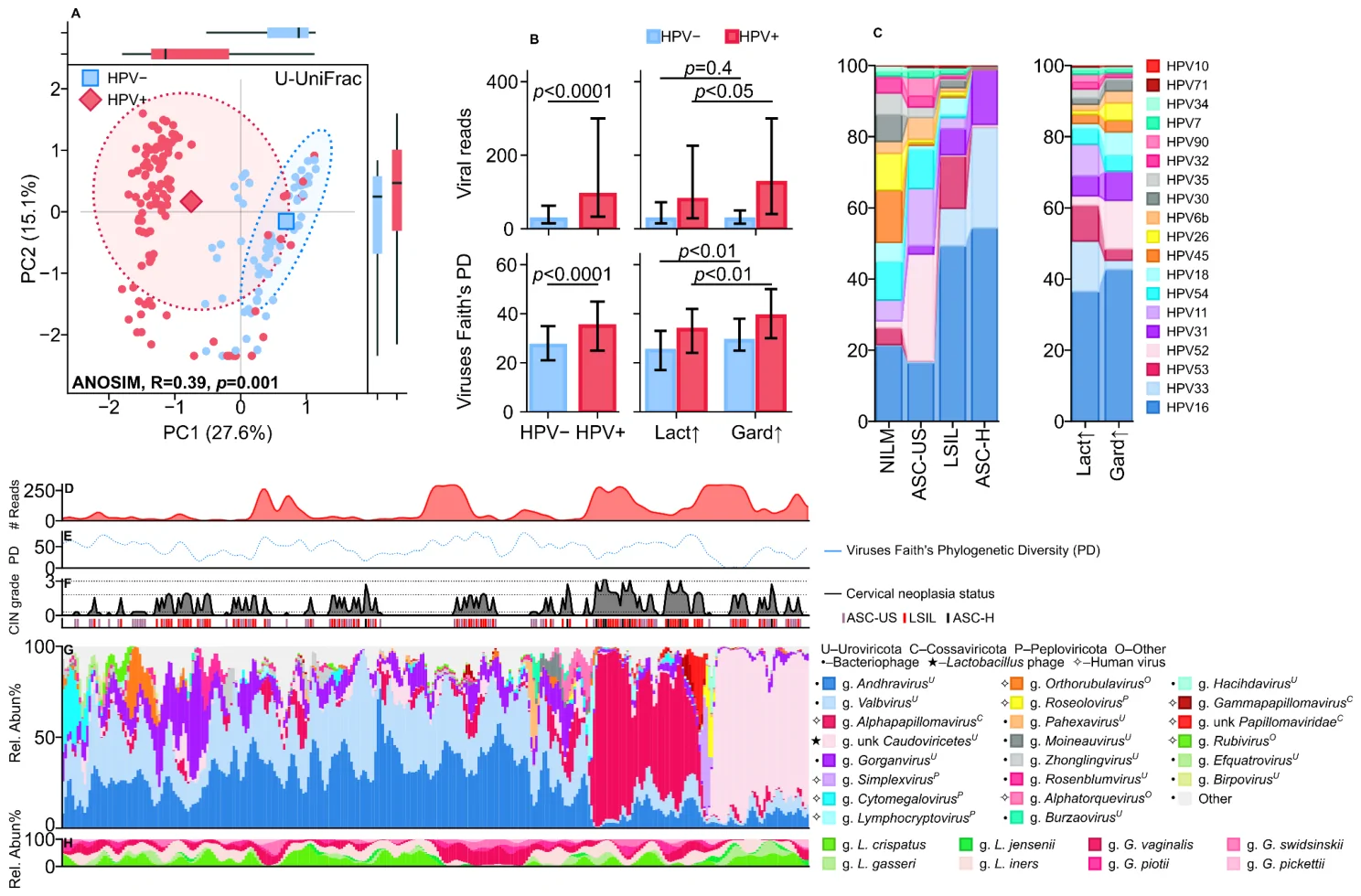
Cervicovaginal virome restructuring associated with HPV infection and bacterial dysbiosis (*n* = 311). (**A**) Unweighted UniFrac PCoA of viral communities by HPV status (ANOSIM R = 0.39, *p* = 0.001). (**B**) Viral load (reads) and α-diversity (Faith’s PD) by HPV, and bacterial community state (Lact↑—*Lactobacillus*; Gard↑—*Gardnerella*). (**C**) HPV genotype composition across cytological grades (NILM, ASC-US, LSIL, ASC-H) and bacterial community state. (**D**–**H**) Per-sample landscape (samples ordered identically in all subplots): (**D**) viral reads number; (**E**) viral Faith’s phylogenetic diversity (PD) index; (**F**) CIN grade and cytology; (**G**) viral genera, annotated by phylum (U/C/P/O) and host (bacteriophage, *Lactobacillus* phage, human virus); (**H**) per sample *Lactobacillus* and *Gardnerella* species relative abundance.

Shotgun metagenomic sequencing of cervicovaginal specimens from 311 participants revealed a multi-kingdom virome with a notable binary polarity relative to HPV status (Figure 1A). Consistent with this restructuring, estimated viral load and phylogenetic α-diversity (Faith’s PD) were higher in HPV+ samples (Figure 1B). Species-level analysis of *Alphapapillomavirus* species across cytological categories revealed a progressive oncogenic gradient (Figure 1C). In NILM HPV+, the composition was taxonomically diverse, including low-risk genotypes (HPV6, HPV11), rare types (HPV26, HPV53), polyomaviruses, and high-risk genotypes. In LSIL and ASC-H categories, the composition shifted toward the *Alphapapillomavirus* 9 clade (HPV16, HPV31, HPV33) [46], the species group responsible for approximately 75% of invasive cervical cancers worldwide [6], with HPV16 occupying a dominant position in ASC-H while low-risk genotypes and polyomaviruses were eliminated.

The per-sample distribution of viral reads, phylogenetic diversity and cervical status are shown in the per-sample landscape of Figure 1D–F. The most prevalent genera of the multi-kingdom virome revealed by shotgun metagenomic sequencing of cervicovaginal specimens from 311 participants were (Figure 1G): *Andhravirus* (prevalence 99%, f. *Rountreeviridae*), *Valbvirus* (96%, f. *Peduoviridae*), *Gorganvirus* (80%, unclassified c. *Caudoviricetes*, unclassified *Caudoviricetes* (35%, unclassified c. *Caudoviricetes*), *Alphapapillomavirus* (33%, f. *Papillomaviridae*), *Zhonglingvirus* (26%, f. *Casjensviridae*), *Rosenblumvirus* (24%, f. *Rountreeviridae*), *Rubivirus* (14%, f. *Matonaviridae*), *Efquatrovirus* (12%, unclassified c. *Caudoviricetes*), *Cytomegalovirus* (12%, f. *Orthoherpesviridae*), *Alphatorquevirus* (9%, f. *Anelloviridae*), *Moineauvirus* (9%, f. *Aliceevansviridae*), *Orthorubulavirus* (9%, f. *Paramyxoviridae*), *Pahexavirus* (7%, unclassified c. *Caudoviricetes*), unclassified *Papillomaviridae* (6%, f. *Papillomaviridae*), *Gammapapillomavirus* (5%, f. *Papillomaviridae*), *Lymphocryptovirus* (3%, f. *Orthoherpesviridae*), *Roseolovirus* (2%, f. *Orthoherpesviridae*), *Simplexvirus* (2%, f. *Orthoherpesviridae*), *Burzaovirus* (1%, f. *Suoliviridae*), *Hacihdavirus* (1%, f. *Intestiviridae*), and *Birpovirus* (1%, f. *Suoliviridae*).

Excluding differential abundance of *Papillomaviridae* (0.0% vs. 9.48% (0.0; 55.56)), the distribution of relative abundance primarily differed between HPV statuses for the family of unclassified *Caudoviricetes*, which mainly encompasses *Lactobacillus* phages. The relative abundance was 17.39% (8.71–40.66) vs. 8.33% (3.63–18.5) (unadjusted *p* < 0.001).

At the genus level, a significant relative reduction was observed for the relative abundance of *Andhravirus* (27.27% (13.57; 37.58) vs. 14.83% (6.9; 36.37), unadjusted *p* = 0.01) tracking after HPV abundance increase; this genus includes *Staphylococcus* epidermidis-infecting, strictly lytic picoviruses [47,48]. A comparable reduction was observed for *Valbvirus* (25.0% [14.29; 37.52] vs. 15.39% [5.99; 30.19], unadjusted *p* < 0.0001), whose closest reference genome is the type species *Vibrio* phage ValB1MD-2. Notably, the *Valbvirus*/*Andhravirus* ratio was lower in HPV+ samples (1.0 [IQR 0.64; 1.45] vs. 0.92 [IQR 0.6; 1.09], *p* < 0.05), indicating a relative compositional shift between these two dominant viral genera.

Finally, a significant relative reduction was also observed for *Gorganvirus* (8.01% (0.46; 14.95) vs. 3.53% (0.71; 10.0), *p* = 0.05), for which the type species *Proteus* phage vB_PmiS-Isfahan serves as reference genome; it is a *Proteus*-infecting siphovirus. It should be noted that the last two genera (*Valbvirus* and *Gorganvirus*) likely represent uncharacterized phages denoted by the closest homology [17].

### 3.7. Lactobacillus (pro)Phages

**Figure 2:**
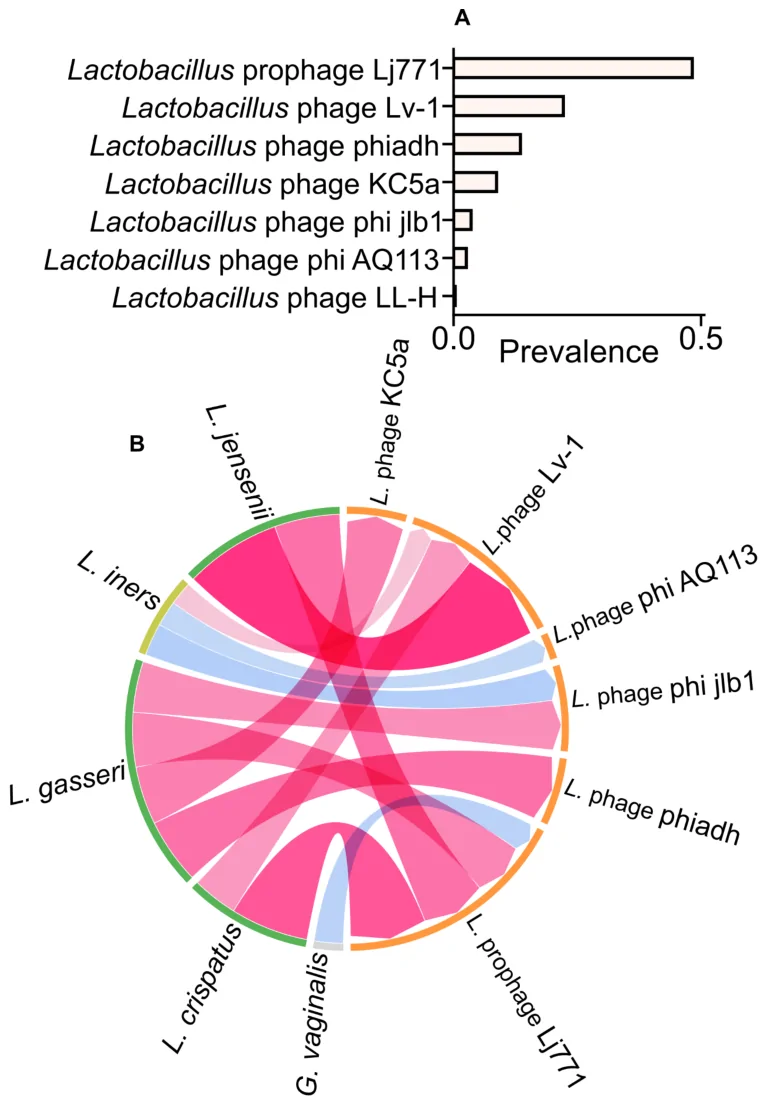
Lactobacillus (pro)phages. (**A**) *Lactobacillus* (pro)phages are prevalent in the cohort, led by prophage Lj771 and phage Lv-1. (**B**) Chord diagram of co-occurrence between *Lactobacillus* (pro)phages and dominant bacterial taxa, showing that (pro)phage carriage tracks with *Lactobacillus*-dominated, protective CSTs. Both wider flows in the chord diagram and more intense red colors indicate higher co-occurrence.

Targeted screening for *Lactobacillus* (pro)phages [49] identified seven taxa across the cohort (Figure 2A). The most prevalent were *Lactobacillus* prophage Lj771 (48%) and *Lactobacillus* phage Lv-1 (20%), followed at progressively lower frequencies by phages phiadh, KC5a, phi jlb1, and phi AQ113, whereas *Lactobacillus* phage LL-H was almost absent.

Co-occurrence analysis (Figure 2B) showed that (pro)phage carriage tracked predominantly with *Lactobacillus*-dominated communities, which are considered protective, chiefly *L. crispatus*, *L. gasseri*, and *L. jensenii*, rather than with *Gardnerella vaginalis*, and in particular with *L. iners*. This pattern links phage presence to *Lactobacillus*-dominated, protective community state types (CSTs).

### 3.8. Functional Landscape of the Cervical Phageome: Lysogeny Balance and Auxiliary Metabolic Genes

Pharokka annotation of phage-encoded genes across nine functional categories revealed that HPV-positive women carried significantly higher absolute gene counts (*p* < 0.001, Table 3); this contrast was strongest between NILM HPV− and NILM HPV+ (*p* < 0.0001). Because NILM HPV-positive samples also yielded higher viral contig counts, differences in individual gene prevalence may partly reflect differences in viral sequence recovery. The proportional composition remained stable, with a single exception: the moron/AMG/host-takeover category showed a significant relative enrichment in HPV+ women (4.66% vs. 2.94%; *p* < 0.05). Neither prophage integration genes (*p* = 0.37) nor lysis genes (*p* = 0.46) differed proportionally. Thus, the category-level analysis did not support a global shift toward either predicted lifecycle state. Subsequent gene-level analyses were therefore interpreted as differences in individual predicted gene prevalence rather than evidence of lifecycle transition.

Stratification by cervical cytology showed that ASC-H/HPV+ women exhibited the highest relative proportion of prophage integration genes (6.03% (3.49; 10.86), twice that of NILM/HPV−) despite comparable contig counts (44.0%, IQR 36.3; 67.3), suggesting selective retention of integration-competent phages in advanced cervical lesions.

Gene-level analysis with FDR correction comparing NILM HPV-negative and NILM HPV-positive samples identified differential prevalence of several predicted genes associated with phage integration, excision, transcriptional regulation, and lysis. Integrase prevalence was 65.7% versus 85.4% (*q* < 0.05), and excisionase prevalence was 2.0% versus 24.4% (*q* < 0.001), respectively. Transcriptional repressor and anti-repressor Ant annotations were also more prevalent in NILM HPV-positive samples (50.5% vs. 78.0% and 37.4% vs. 63.4%, respectively; *q* < 0.05). Among lysis-associated genes, amidase differed between groups (1.0% vs. 24.4%; *q* < 0.001), whereas endolysin and holin did not (Table 4). These results indicate differences in predicted lifecycle-associated gene prevalence but do not establish active prophage induction or lifecycle switching.

Within the moron/AMG/host-takeover category, three functionally coherent gene clusters were enriched in NILM/HPV+ (Table 5). Cluster A (nucleotide metabolism) comprised thymidylate synthase (0% vs. 22.0%; *q* < 0.001), dUTPase (3.0% vs. 19.5%; *q* < 0.01), and dihydrofolate reductase (2.0% vs. 14.6%; *q* < 0.05)—phage-encoded orthologs of enzymes targeted by 5-fluorouracil and methotrexate that provide autonomous dNTP supply for phage replication and may additionally alter the nucleotide pool in HPV-proliferating cervical epithelial cells. Cluster B (anti-restriction and DNA modification) included DarB antirestriction protein (2.0% vs. 24.4%; *q* < 0.01), QueC queuosine biosynthesis protein (0% vs. 9.8%; *q* < 0.01), and DNA methyltransferase (46.5% vs. 75.6%; *q* < 0.01)—genes that shield phage DNA from restriction–modification systems and can epigenetically reprogram host bacterial gene expression. Cluster C (toxin–antitoxin and host takeover) encompassed the HicB addiction module (27.3% vs. 58.5%; *q* < 0.01), abortive-infection resistance protein (6.1% vs. 29.3%; *q* < 0.01), and ABC transporter (20.2% vs. 53.7%; *q* < 0.01)—genes that enforce bacterial dependence on resident phages and commandeer host metabolism.

### 3.9. Community-State-Type Stratification Reveals Contrasting Predicted Phage Functional Profiles

**Figure 3:**
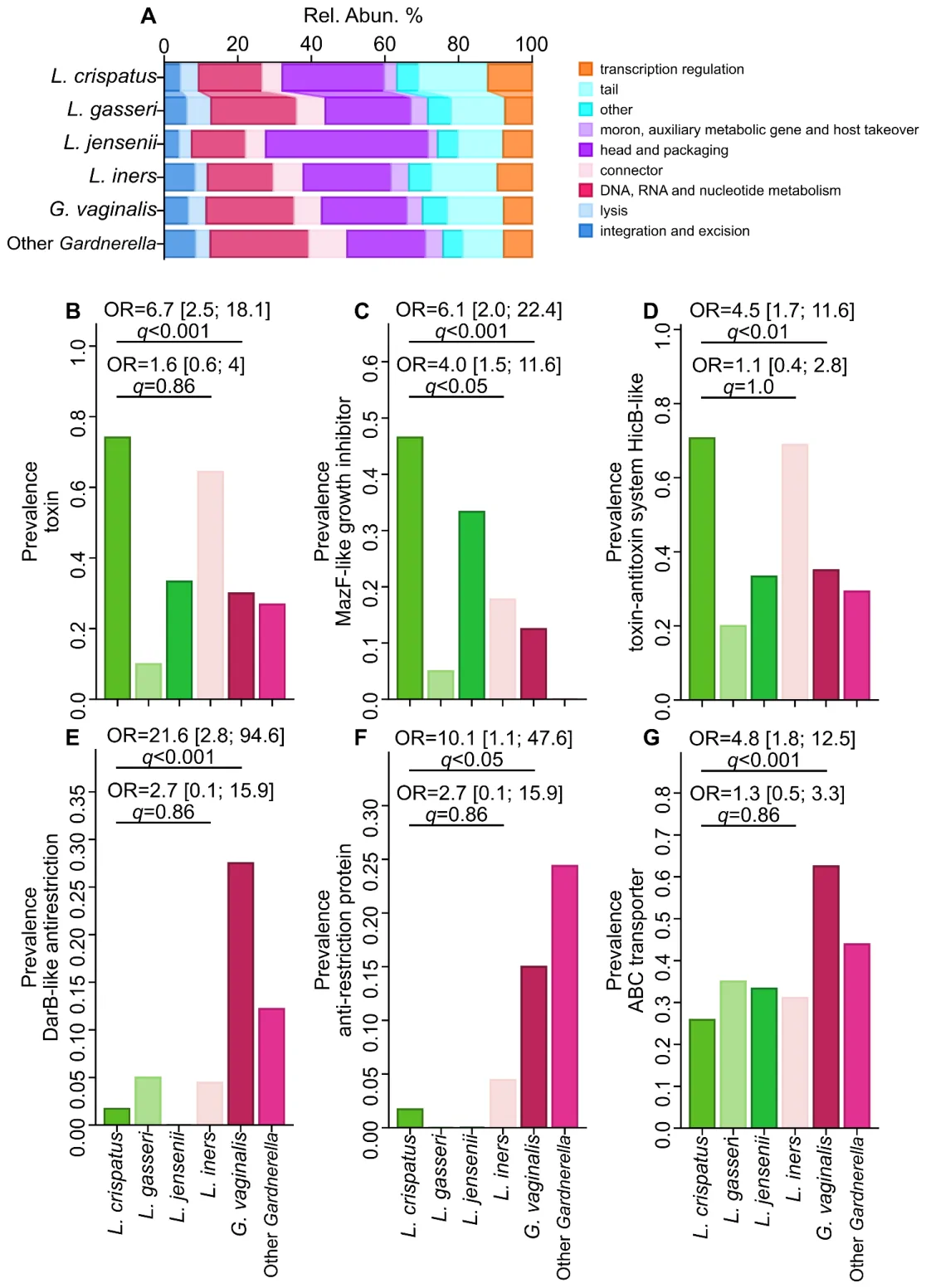
Predicted phage functional profiles across cervicovaginal community state types (CSTs). (**A**) Relative composition of nine PHROG functional categories of phage-encoded genes across dominant cervicovaginal taxa (*L. crispatus*, *L. gasseri*, *L. jensenii*, *L. iners*, *G. vaginalis*, other *Gardnerella* spp.). (**B**–**D**) Prevalence of toxin–antitoxin module genes enriched in Lactobacillus-dominated communities: (**B**) toxin, (**C**) MazF-like growth inhibitor, (**D**) HicB-like addiction module. (**E**–**G**) Prevalence of counter-defense and host-takeover genes enriched in *Gardnerella*-dominated communities: (**E**) DarB-like antirestriction protein, (**F**) anti-restriction protein, (**G**) ABC transporter. In (**B**–**G**), bars show prevalence by dominant taxon; odds ratios contrast CST I (*L. crispatus*) vs. CST III (*L. iners*) and CST I vs. CST IV (*Gardnerella*. q-values, Benjamini–Hochberg FDR.

The functional shifts described above were defined along the HPV axis. Because the phageome tracks its bacterial hosts, we next examined how the same predicted gene repertoire varied according to bacterial community state type. We therefore re-stratified phage functional genes by community state type, contrasting CST I (*L. crispatus*) with CST III (*L. iners*) and with CST IV (*Gardnerella vaginalis*) (Figure 3A).

This host-axis analysis revealed contrasting predicted functional profiles that partially overlapped with those observed in the HPV-stratified analysis. *Lactobacillus*-dominated communities were selectively enriched for intracellular toxin–antitoxin (TA) modules: the generic toxin category (OR = 6.7 [2.5; 18.1], *q* < 0.001), the MazF-like growth inhibitor (OR = 6.1 [2.0; 22.4], *q* < 0.001), and the HicB-like addiction module (OR = 4.5 [1.7; 11.6], *q* < 0.01) were all over-represented in CST I relative to CST IV (Figure 3B–D). The MazF-like enrichment was also significant within the *Lactobacillus* clade itself (CST I vs. CST III (*L. iners*): OR = 4.0 [1.5; 11.6], *q* < 0.05), indicating a variation even among lactobacilli. Conversely, *Gardnerella*-dominated communities were enriched for an outward-facing anti-restriction and host-takeover arsenal: the DarB-like anti-restriction protein (OR = 21.6 [2.8; 94.6], *q* < 0.001), a second anti-restriction protein (OR = 10.1 [1.1; 47.6], *q* < 0.05), and an ABC transporter (OR = 4.8 [1.8; 12.5], *q* < 0.001) (Figure 3E–G). The abortive-infection resistance functions enriched along the HPV axis (Cluster C) were not strictly community-state-type-specific, although their prevalence appeared to decline in *L. iners*–dominated communities, suggesting that the HPV and host axes capture overlapping but non-identical signals.

We have further performed adjusted analyses for all PHROG categories, adjusting for age, parity, marital status, BMI, HPV status, cervical cell status, and CST. For cell status, NILM was the reference value; for CST, the *L. crispatus* was the reference. We performed this analysis using MaAsLin3 v1.2.0 [50], using only the prevalence modeling mode (evaluate_only = “prevalence”). Multiple testing adjustment was performed using FDR BH, and associations were considered significant at *q* < 0.05. This analysis detected several significant associations, consistent with the prior unadjusted analyses. In particular, in the PHROG “lysis” category, the term “amidase” prevalence was found to be significantly higher in HPV+ patients. Similarly to the main analysis, holin was not differentially prevalent. In the “host-takeover” category, significant associations with CST were reproduced. In particular, CST Gardnerella was found to be associated with elevated “DarB anti-restriction”, “ABC transporter”, and “anti-restriction protein” terms, while crispatus was found to harbor more “toxin–antitoxin system HicB”, “toxin”, and “MazF-like growth inhibitor” terms, both in comparison to CST Gardnerella and CST iners. Additionally, in the “integration” category, the “excisionase and transcriptional regulator” term was found to be more prevalent in CST Gardnerella. These analyses can be found in the Supplementary Materials.

These findings indicate contrasting predicted phage functional repertoires across bacterial community states: protective *Lactobacillus* communities carry a TA-dominated, lysogeny-compatible repertoire, while *Gardnerella*-associated dysbiosis carries a restriction-escape repertoire. Two genes that contributed to the HPV-defined Cluster B/C signal were resolved by this analysis into distinct host associations: DarB tracked with *Gardnerella* dominance, identifying it as a dysbiosis-associated restriction-escape factor, whereas the HicB module tracked with *Lactobacillus* dominance.

## 4. Discussion

This study provides an extensive shotgun metagenomic characterization of the cervicovaginal virome across a range of HPV-associated cervical cytological categories in a Central Asian population.

The high prevalence of CST IV-B (38%) exceeds proportions typically reported in European and East Asian populations [20,21], potentially reflecting population-specific differences. The genotype distribution, with a notably high prevalence of α-9 clade genotypes (59.7%), more closely resembles East and Southeast Asian patterns than European ones. A recent prospective study from our center confirmed that vaginal microecological disorders are significantly associated with HPV infection risk in Kazakhstani women [22], underscoring the need for population-specific microbiome and virome data to inform regional screening strategies.

Our analysis indicates HPV-associated virome restructuring at two levels: (i) a narrowing of the eukaryotic virome toward the oncogenic *Alphapapillomavirus 9* clade across more abnormal cytological categories, and (ii) functional remodeling of the phageome, reflected by differences in the prevalence of lifecycle-associated genes and enrichment of auxiliary metabolic and host-interaction genes related to nucleotide metabolism, DNA modification, and bacterial defense–counter-defense functions. In particular, differences in integrase, excisionase, transcriptional repressor, anti-repressor, and lysis-associated annotations suggest variation in the predicted lifecycle-associated genetic repertoire of the phageome, but do not establish active prophage induction or lysogeny–lysis switching. These results extend the current understanding of the cervicovaginal microenvironment, which has been characterized predominantly at the bacteriome level [2], by identifying a parallel virome axis associated with both bacterial community composition and cervical cytological status.

To our knowledge, no comparable cervicovaginal virome dataset has previously been available for this region.

The CST-stratified analysis identified differences in predicted phage functional repertoires across bacterial community states. Toxin–antitoxin-associated annotations were more prevalent in *L. crispatus*-dominated CST I, whereas anti-restriction-related annotations were more prevalent in *Gardnerella*-dominated communities. These patterns are consistent with the close ecological relationship between bacteriophages and their bacterial hosts. However, overall CST distribution did not differ significantly across the seven clinical groups in the present cohort. Moreover, HPV status and bacterial community composition were not fully independent, because HPV-positive samples showed lower *Lactobacillus* and higher *Gardnerella* abundance. Therefore, the HPV- and CST-associated functional patterns should be regarded as partially overlapping ecological associations unless independence is supported by adjusted analyses.

Several differentially represented annotations were associated with nucleotide metabolism, including thymidylate synthase, dUTPase, and dihydrofolate reductase, whereas other annotations included DarB-like anti-restriction proteins, DNA methyltransferases, and toxin–antitoxin-related genes. These functions have established roles in phage biology and bacterial host interactions and therefore provide plausible hypotheses regarding phage functional variation across cervicovaginal ecological states. However, the present analysis measured predicted gene presence rather than transcription, protein activity, or metabolite concentrations. Accordingly, the data do not demonstrate that these genes modify nucleotide availability, bacterial transcription, cervical epithelial metabolism, or host epigenetic regulation in vivo.

The relative abundances of both *Valbvirus* and *Andhravirus* differed by HPV status, and the *Valbvirus*/*Andhravirus* ratio also varied between groups. These findings indicate a compositional difference between two prominent phage taxa rather than a demonstrated biological transition from one viral taxon or lifecycle state to another. Because the closest *Valbvirus* reference in this analysis is the *Vibrio phage ValB1MD-2*, *Valbvirus* should not be described as a *Lactobacillus*-associated phage without independent host-assignment evidence.

**Figure 4:**
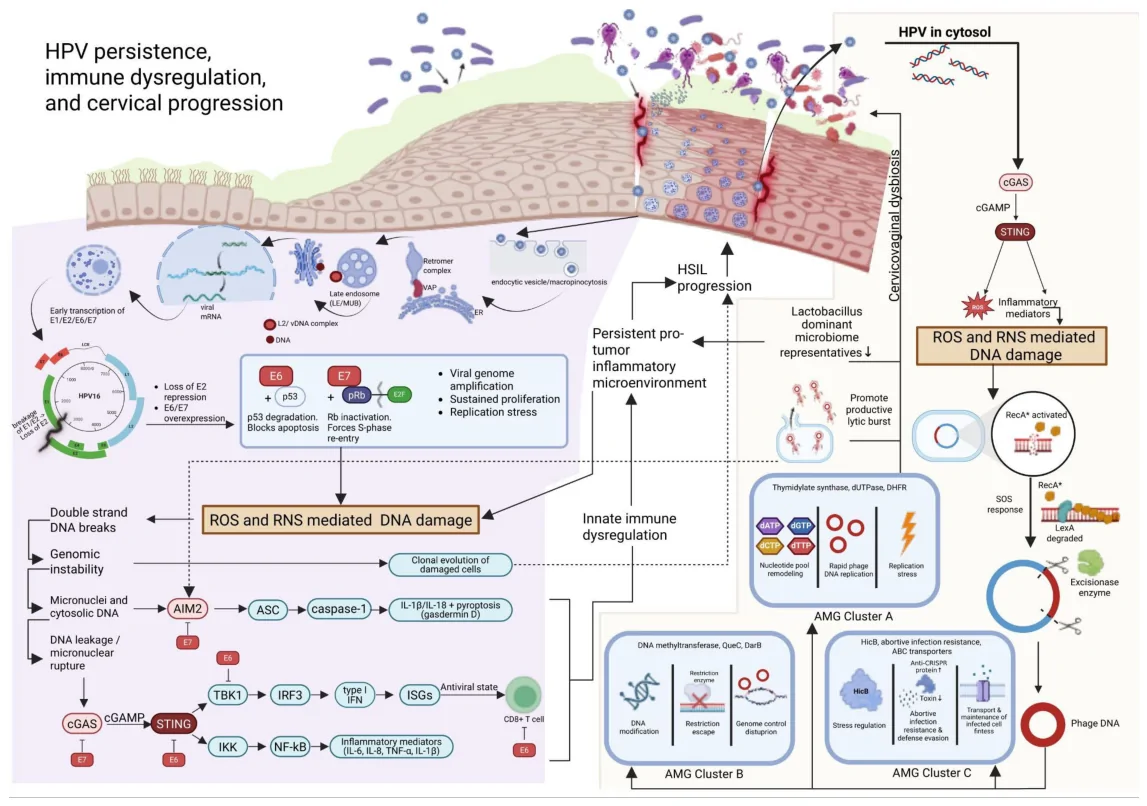
Proposed three-stage model of HPV-driven virome restructuring and the self-reinforcing virome–dysbiosis cycle in the cervicovaginal niche. The hypothesis-generating model integrates associations observed in the present cross-sectional study with mechanisms proposed in previous literature. Schematic integration of taxonomic and functional data into a tripartite HPV–bacteriome–phageome framework. **Stage 1:** E6/E7 overexpression suppresses cGAS–STING and AIM2 signaling and depletes host antimicrobial peptides, collapsing *Lactobacillus*-associated phages and shifting phageome dominance toward lytic-dominated prophage communities. **Stage 2:** cGAS–STING activation and ROS/RNS-mediated DNA damage trigger the bacterial SOS response and excisionase-mediated prophage induction from *Lactobacillus* genomes. **Stage 3:** Released lytic phages carry three AMG clusters: nucleotide-pool remodeling (Cluster A), restriction escape and epigenetic reprogramming (Cluster B), and host-fitness control (Cluster C), sustaining a self-reinforcing virome–dysbiosis cycle that drives HSIL progression.

Integrating the taxonomic, functional, and clinical data, we outline a possible three-stage model of HPV-associated virome restructuring (Figure 4). The model should not be interpreted as a temporal sequence demonstrated by the present cross-sectional data. Stage 1: HPV immune evasion (E6/E7-mediated suppression of defense peptides) depletes *Lactobacillus* populations and their nutrient supply, collapsing *Lactobacillus*-associated phages and shifting phageome dominance toward lytic-dominated prophage communities. Stage 2: HPV-driven cGAS–STING activation triggers SOS-dependent prophage induction, releasing lytic phages from surviving *Lactobacillus* genomes; the excisionase/integrase dissociation and concurrent repressor/Ant enrichment documented in our data provide the molecular evidence for this step. Stage 3: The released lytic phages carry AMGs (thymidylate synthase, dUTPase, DHFR, DNA methyltransferase) capable of modulating the nucleotide pool and epigenetic landscape of the cervical microenvironment, potentially creating conditions that favor genomic instability and lesion progression. This model extends the established HPV–bacteriome axis to a tripartite HPV–bacteriome–phageome framework in which each component may amplify the others.

Despite differentially abundant viral functional repertoires in HPV-negative and HPV-positive samples, it should be kept in mind that viral load varied significantly by HPV status, with positive samples having higher loads. Thus, the observed differences can and should be explained, in part, through the lens of viral load. Nonetheless, our analysis provides a valuable description of the viral functional repertoire in relation to various functional and taxonomic outcomes.

### Limitations

This study has several limitations. The cross-sectional design precludes causal inference; longitudinal validation is needed. Shotgun sequencing without viral enrichment limits sensitivity for low-abundance taxa, and the DNA-based protocol did not capture the RNA virome. The extraction control lacked viral components, precluding direct validation of virus recovery. The small ASC-H subgroup (*n* = 10) and absence of HSIL/carcinoma groups restrict extrapolation to advanced disease. Attribution of specific AMGs to individual phage taxa would require long-read or Hi-C sequencing. Unmeasured confounders (smoking, contraception, vaginal pH) may have influenced virome composition. The HPV and community-state-type axes are partially confounded, since HPV+ status is associated with *Gardnerella* abundance, so gene associations identified along one axis cannot be directly distinguished from the other. Finally, it should be kept in mind that metagenomic assignment can be misleading for small fractions and for less-studied ecological niches, such as the cervical community compared to the human gut. Thus, the abundances and prevalence of less prominent taxa (non-*Papillomaviridae*) detected and reflected in the present study may differ from the true underlying abundances, warranting future targeted validation.

## 5. Conclusions

HPV status and cervical cytological category were associated with differences in cervicovaginal viral community composition and predicted phage functional gene prevalence. HPV-positive samples showed altered representation of dominant viral taxa, including *Andhravirus* and *Valbvirus*, while the oncogenic *Alphapapillomavirus 9* clade was increasingly represented across more abnormal cytological categories. Predicted lifecycle-associated, nucleotide-metabolism, DNA-modification, anti-restriction, and toxin–antitoxin-related annotations also differed between NILM HPV-positive and NILM HPV-negative samples. Stratification by bacterial community state type further identified contrasting phage functional profiles, with toxin–antitoxin-associated annotations more prevalent in *Lactobacillus crispatus*-dominated communities and anti-restriction-associated annotations more prevalent in Gardnerella vaginalis-dominated communities. Together, these findings support further investigation of cross-kingdom relationships among HPV, bacterial communities, and the cervicovaginal phageome. However, the present cross-sectional metagenomic data do not demonstrate active prophage induction, temporal virome progression, or direct effects of predicted phage genes on cervical host metabolism or lesion development. Longitudinal and experimentally validated studies are required to establish the directionality and biological significance of these associations. Viral load varied significantly by HPV status, and the observed differences should be considered in this context.

## Figures and Tables

**Table 1:** Clinical and demographic characteristics of study participants by groups.

Parameter	NILM HPV−, (*n* = 103)	NILM HPV+, (*n* = 42)	ASC-US HPV− (*n* = 39)	ASC-US HPV+ (*n* = 36)	LSIL HPV− (*n* = 20)	LSIL HPV+ (*n* = 61)	ASC-H HPV+ (*n* = 10)	*p*-Value
**Demographic indicators**								
Age, years, Md (IQR)	34.13 (31.4; 39.51)	34.56 (28.02; 38.45)	36.52 (31.8; 39.99)	34.74 (29.11; 38.52)	37.39 (34.5; 41.04)	32.69 (27.88; 36.5)	36.31 (31.39; 39.98)	*p* = 0.11 ^a^
BMI, kg/m^2^, Md (IQR)	22.68 (20.55; 25.24)	23.55 (20.58; 28.22)	23.34 (20.43; 25.28)	21.62 (20.4; 25.85)	23.94 (21.04; 27.09)	22.77 (20.07; 24.92)	23.31 (20.23; 30.11)	*p* = 0.67 ^a^
**Reproductive history**								
Age at menarche, years, Md (IQR)	13.0 (13.0; 14.0)	13.0 (13.0; 14.0)	14.0 (13.0; 15.0)	13.0 (12.0; 13.0)	13.0 (13.0; 15.0)	13.0 (13.0; 14.0)	13.5 (13.0; 14.0)	*p* = 0.4 ^a^
History of pregnancy, *n* (%)	86 (83%)	28 (67%)	27 (69%)	24 (67%)	17 (85%)	41 (67%)	9 (90.0%)	*p* = 0.07 ^b^
Pregnancies, Md (IQR)	2 (1; 4)	1.5 (0; 3)	2 (0; 4)	2 (0; 3)	2.5 (1; 3.25)	1 (0; 3)	1 (1; 2)	*p* = 0.10 ^a^
Child births, Md (IQR)	2 (1; 3)	1 (0; 2)	2 (0; 3)	1.5 (0; 2)	2 (1; 3)	1 (0; 2)	1 (1; 2)	***p*** **< 0.05 ^a^**
Number of sexual partners, Md (IQR)	1 (1; 1)	1 (1; 2)	1 (1; 2)	1(1; 1)	1 (1; 1)	1 (1; 2)	1 (1; 1.75)	*p* = 0.49 ^a^
Marital status, married, *n* (%)	93 (90%)	28 (67%)	28 (72%)	25 (69%)	14 (70%)	44 (72%)	6 (60%)	***p*** **< 0.01 ^b^**
**HPV status and genotyping**								
HPV+, PCR, *n* (% of 145)	—	18 (12.4%)	—	28 (37.3%)	—	56 (70.4%)	10 (100%)	***p*** **< 0.0001 ^b^**
HPV+, MNGS, *n* (%)	—	41 (28.3%)	—	25 (33.3%)	—	46 (56.8%)	8 (80%)	***p*** **< 0.0001 ^b^**
Viral load, PCR HPV+, ×10^5^		4.75 (4.22; 5.95)		3.75 (1.6; 5.75)		4.7 (2.1; 6.2)	6.5 (5.12; 8.18)	*p* = 0.1 ^a^
Viral load, MNGS HPV+, relative abundance (%)		5.88 (1.26; 29.64)		17.22 (6.85; 42.11)		53.49 (10.28; 75.49)	86.92 (78.79; 94.67)	***p* < 0.0001** ^a^
Genotype Prevalence, PCR, (%)	—	HPV16 (27.8%), HPV45 (27.8%), HPV52, (22.2%), HPV39 (16.7%)	—	HPV16 (21.4%), HPV52, (21.4%), HPV33, (14.3%) HPV39 (10.7%)	—	HPV16 (33.9%), HPV52 (19.6%), HPV33 (19.6%), HPV59 (14.3%)	HPV16 (40.0%), HPV58 (30.0%), HPV33 (30.0%), HPV59 (20.0%)	*p* = 0.52 ^b^†
Genotype Prevalence, MNGS, (%)	—	α-9 group (31.7%) α-7 group (29.3%) α-6 group (19.5%) α-3 group (12.2%) [HPV16 (14.6%)]	—	α-9 group (64.0%) α-7 group (16.0%) α-6 group (4.0%) α-3 group (24.0%) [HPV16 (32.0%)]	—	α-9 group (76.1%) α-7 group (19.6%) α-6 group (23.9%) α-3 group (19.6%) [HPV16 (52.2%)]	α-9 group (100%) α-7 group (0%) α-6 group (12.5%) α-3 group (12.5%) [HPV16 (62.5%)]	***p* < 0.0001** ^b^††
**Dominant bacterial genera distribution**								
*Lactobacillus*, Md (IQR)	27.39 (2.4; 81.43)	17.05 (0.11; 83.48)	71.85 (12.45; 94.92)	32.88 (0.16; 93.83)	21.09 (6.5; 79.88)	12.01 (0.76; 69.6)	9.66 (1.93; 48.05)	*p* = 0.11 ^b^ *p* < 0.05 ^c^†††
*Gardnerella*, Md (IQR)	8.28 (0.41; 60.73)	25.57 (5.25; 73.36)	1.77 (0.31; 29.67)	15.43 (1.1; 67.02)	15.38 (0.74; 67.41)	18.22 (0.83; 84.09)	59.25 (0.54; 80.11)	*p* = 0.14 ^b^ *p* < 0.05 ^c^†††
CST								
CST I (*L. crispatus)*, *n* (%)	22 (21%)	6 (14%)	12 (31%)	13 (36%)	4 (20%)	11 (18%)	1 (10%)	*p* = 0.57 ^b^
CST II (*L. gasseri*), *n* (%)	8 (8%)	3 (7%)	7 (18%)	1 (3%)	2 (10%)	5 (8%)	2 (20%)	
CST III (*L. iners*), *n* (%)	28 (27%)	13 (31%)	10 (26%)	5 (14%)	6 (30%)	21 (34%)	3 (30%)	
CST V (*L. jensenii*), *n* (%)	4 (4%)	2 (5%)	1 (3%)	2 (6%)	1 (5%)	0 (0%)	0 (0%)	
CST IV-B (1) (*Gardnerella* spp., excluding *G. vaginalis*), *n* (%)	22 (21%)	9 (21%)	6 (15%)	5 (14%)	4 (20%)	14 (23%)	3 (30%)	
CST IV-B (2) (*Gardnerella vaginalis*), *n* (%)	19 (18%)	9 (21%)	3 (8%)	10 (28%)	3 (15%)	10 (16%)	1 (10%)	

^a^ Kruskal–Wallis H-test. ^b^ Chi2. ^c^ Mann–Whitney U test. † comparing HPV16 prevalence over other HPV types. †† comparing α-9 prevalence over other HPV groups/types. ††† comparing HPV-negative and HPV-positive samples. Statistically significant results are highlighted in bold. α-9: https://www.ncbi.nlm.nih.gov/Taxonomy/Browser/wwwtax.cgi?lvl=0&id=337041 (accessed on 1 June 2026).

**Table 2:** Consistency of MNGS HPV detection with PCR.

Consistency Status	*n*	%	Interpretation
PCR+/MNGS+	84	74.3%	Both methods confirm HPV infection (relative to PCR)
PCR−/MNGS−	162	81.8%	Both methods do not detect HPV
PCR+/MNGS−	29	25.7%	PCR is positive, MNGS did not detect the virus; the viral load is likely below the MNGS detection threshold
PCR−/MNGS+	36	18.2%	MNGS detected an HPV genotype missing from the targeted PCR panel; expanded coverage of the metagenomic approach

Note: PCR—targeted PCR genotyping; MNGS—shotgun metagenomic sequencing. Percentages for PCR-positive categories are calculated among all PCR-positive samples (*n* = 113), whereas percentages for PCR-negative categories are calculated among all PCR-negative samples (*n* = 198). Overall agreement = 79.1%; positive percent agreement = 74.3%; negative percent agreement = 81.8%; Cohen’s κ = 0.55.

**Table 3:** Viral genes profile.

Parameter	NILM HPV−, (*n* = 103)	NILM HPV+, (*n* = 42)	ASC-US HPV− (*n* = 39)	ASC-US HPV+ (*n* = 36)	LSIL HPV− (*n* = 20)	LSIL HPV+ (*n* = 61)	ASC-H HPV+ (*n* = 10)	*p*-Value
**Abs. number of viral hallmark genes**								
Num. viral hallmark genes, Md (IQR)	40.0 (19.0; 63.0)	101.0 (34.0; 206.0)	66.0 (30.75; 106.75)	75.0 (34.5; 138.0)	34.0 (8.0; 72.5)	49.0 (14.5; 103.25)	58.5 (28.25; 84.0)	***p* < 0.001 ^a^†** ***p* < 0.0001 ^a^††**
Contig count, Md (IQR)	48.5 (IQR: 33.25; 64.0)	74.5 (IQR: 44.25; 139.25)	47.0 (IQR: 40.0; 58.0)	70.5 (IQR: 51.5; 87.0)	48.0 (IQR: 40.75; 63.75)	53.0 (IQR: 41.0; 73.0)	44.0 (IQR: 36.25; 67.25)	***p* < 0.001 ^a^††**
**Relative abundance of viral genes per functional category**								
Integration and excision, Md (IQR)	3.7 (0.0; 7.6)	4.17 (2.33; 9.33)	4.25 (3.08; 6.17)	4.31 (2.42; 6.51)	3.51 (0.0; 8.37)	3.23 (0.0; 6.42)	6.03 (3.49; 10.86)	*p* = 0.37 ^a^† *p* = 0.39 ^a^††
Lysis, Md (IQR)	4.08 (0.0; 6.4)	4.69 (2.35; 6.25)	5.77 (3.09; 7.69)	4.23 (2.67; 5.76)	4.98 (0.0; 6.96)	4.15 (0.0; 6.3)	4.91 (1.7; 6.74)	*p* = 0.46 ^a^† *p* = 0.38 ^a^††
Moron, auxiliary metabolic gene and host takeover	2.78 (0.0; 6.2)	5.14 (3.17; 6.21)	3.49 (1.26; 5.24)	3.38 (2.32; 5.65)	2.74 (0.0; 5.72)	2.9 (0.0; 5.73)	2.84 (0.0; 6.0)	*p* = 0.27 ^a^† ***p* < 0.05 ^a^††**

^a^ Kruskal–Wallis. † comparing all groups. †† comparing NILM HPV− vs. NILM HPV+. Statistically significant results are highlighted in bold.

**Table 4:** Phage lifecycle (lysogeny–lysis) genes in NILM HPV-positive women (*n* = 42) versus NILM HPV-negative controls (*n* = 103).

Gene (Functional Role)	Prevalence NILM/HPV− vs. HPV+	OR	95% CI	*Q*-Value
**Lysogeny/excision**				
Integrase (lysogeny maintenance)	65.7% vs. 85.4%	3.1	1.1; 9.7	*q* < 0.05
Excisionase and transcriptional regulator	2.0% vs. 24.4%	15.6	3.0; 151.0	*q* < 0.001
**Lysogeny control**				
Transcriptional repressor (CI-like)	50.5% vs. 78.0%	3.5	1.4; 9.1	*q* < 0.05
Anti-repressor (Ant)	37.4% vs. 63.4%	2.9	1.3; 6.7	*q* < 0.05
**Lysis**				
Amidase	1.0% vs. 24.4%	31.6	4.1; 1385.3	*q* < 0.001
Endolysin	64.6 vs. 80.5	2.3	0.9; 6.3	*q* = 0.11
Holin	not differentially prevalent	1.8	0.8; 4.3	*q* = 0.22

**Notes.** Prevalence represents the proportion of samples in which each predicted gene annotation was detected. Odds ratios (ORs) and 95% confidence intervals (CIs) were calculated from sample-level presence/absence data using two-sided Fisher’s exact tests, with Benjamini–Hochberg false-discovery-rate correction. These annotations are associated with phage lifecycle functions but do not constitute direct evidence of active prophage induction or lysogeny–lysis switching.

**Table 5:** Candidate viral auxiliary metabolic and host-interaction gene annotations differing between NILM HPV-positive (*n* = 42) and NILM HPV-negative (*n* = 103) samples.

Gene (PHROG Annotation)	Prevalence NILM/HPV− vs. HPV+	OR	95% CI	*Q*-Value
**Cluster A (nucleotide metabolism)**				
Thymidylate synthase	0.0% vs. 22.0%	30.3	3.9; 1327.6	*q* < 0.001
dUTPase	3.0% vs. 19.5%	7.8	1.7; 47.2	*q* < 0.05
Dihydrofolate reductase	2.0% vs. 14.6%	8.3	1.4; 86.4	*q* < 0.05
**Cluster B (anti-restriction/DNA modification)**				
DarB antirestriction protein	2.0% vs. 24.0%	15.6	3.0; 150.9	*q* < 0.01
QueC (queuosine biosynthesis)	0.0% vs. 9.8%	23.9	1.3; 454.4	*q* < 0.01
DNA methyltransferase	46.5% vs. 75.6%	3.6	1.5; 9.0	*q* < 0.01
**Cluster C (toxin–antitoxin/host takeover)**				
HicB addiction module	27.3% vs. 58.5%	3.8	1.6; 8.7	*q* < 0.01
Abortive-infection resistance protein	6.1% vs. 29.3%	6.4	2.0; 22.4	*q* < 0.01
ABC transporter	20.2% vs. 53.7%	4.6	1.9; 10.8	*q* < 0.01

Notes. Prevalence denotes the proportion of samples in which the gene was detected. Counts were derived from per-group prevalence within the gene-level differential analysis. Odds ratios (OR) with 95% confidence intervals (CI), two-sided Fisher exact FDR-BH corrected p-values quantify enrichment in HPV+ samples; Cluster A enzymes are the molecular targets of 5-fluorouracil and methotrexate; Cluster B genes mediate restriction escape and epigenetic modification; Cluster C genes enforce host dependence on resident phages. FDR, false discovery rate; PHROG, Prokaryotic Virus Remote Homologous Groups.

## Data Availability

Raw metagenomic sequencing data have been deposited in NCBI under BioProject accession number PRJNA1337009 (https://www.ncbi.nlm.nih.gov/bioproject/?term=PRJNA1337009, accessed on 2 September 2026). The analysis code is publicly available at https://github.com/VeaLi/kraken-analysis-HPV-and-virome (accessed on 2 September 2026), comprising documented Jupyter notebooks for the Kraken2 viral-fraction processing and the geNomad and pharokka annotation steps. The STROBE and STORMS checklists are included as Supplementary Tables S1 and S2.

## Supplementary Materials

The following supporting information can be downloaded at: https://www.mdpi.com/article/10.3390/v18091036/s1, Figure S1: Study flowchart: participant enrollment, HPV verification, sequencing quality control, and final stratification into seven clinical groups (cervicovaginal virome cohort, Kazakhstan); Table S1: STROBE Statement-Checklist of items that should be included in reports of cross-sectional studies; Table S2: STORMS checklist. Strengthening The Organization and Reporting of Microbiome Studies: reporting checklist; Table S3: Classification of human papillomavirus (HPV) genotypes into Alphapapillomavirus species-level groups (α-groups) according to NCBI Taxonomy and the ICTV Papillomaviridae framework.

## Abbreviations

The following abbreviations are used in this manuscript:

AMG	Auxiliary metabolic gene
ANOSIM	Analysis of similarities
ASC-H	Atypical squamous cells, cannot exclude high-grade squamous intraepithelial lesion
ASC-US	Atypical squamous cells of undetermined significance
BMI	Body mass index
bp	Base pairs
CI	Confidence interval
CST	Community state type
Ct	Cycle threshold
DHFR	Dihydrofolate reductase
DNA	Deoxyribonucleic acid
FDR	False discovery rate
HPV	Human papillomavirus
HPV−	HPV-negative
HPV+	HPV-positive
IQR	Interquartile range
LSIL	Low-grade squamous intraepithelial lesion
Md	Median
MNGS	Metagenomic next-generation sequencing / metagenomic sequencing
NCBI	National Center for Biotechnology Information
NILM	Negative for intraepithelial lesion or malignancy
OR	Odds ratio
PCoA	Principal coordinates analysis
PCR	Polymerase chain reaction
PHROGs	Prokaryotic Virus Remote Homologous Groups
RNS	Reactive nitrogen species
ROS	Reactive oxygen species
STORMS	Strengthening The Organization and Reporting of Microbiome Studies
STROBE	Strengthening the Reporting of Observational Studies in Epidemiology
TA	Toxin–antitoxin
α-3	Alphapapillomavirus 3
α-5	Alphapapillomavirus 5
α-6	Alphapapillomavirus 6
α-7	Alphapapillomavirus 7
α-9	Alphapapillomavirus 9
α-10	Alphapapillomavirus 10
