# Supplementary material for: Cervicovaginal Virome Restructuring Associated with HPV Status, Bacterial Dysbiosis, and Cervical Cytological Abnormalities
Source: Viruses. 2026 Sep 19;18(9):1036. doi: 10.3390/v18091036 (PMC13611935; doi:10.3390/v18091036)
Supplement: Supplementary file 1 [file viruses-18-01036-s001.zip › Supplementary_Figure_S1.pdf]

Supplementary Figure S1.

Study flowchart: participant enrollment, HPV verification, sequencing quality control, and final stratification into seven clinical groups (cervicovaginal virome cohort, Kazakhstan).

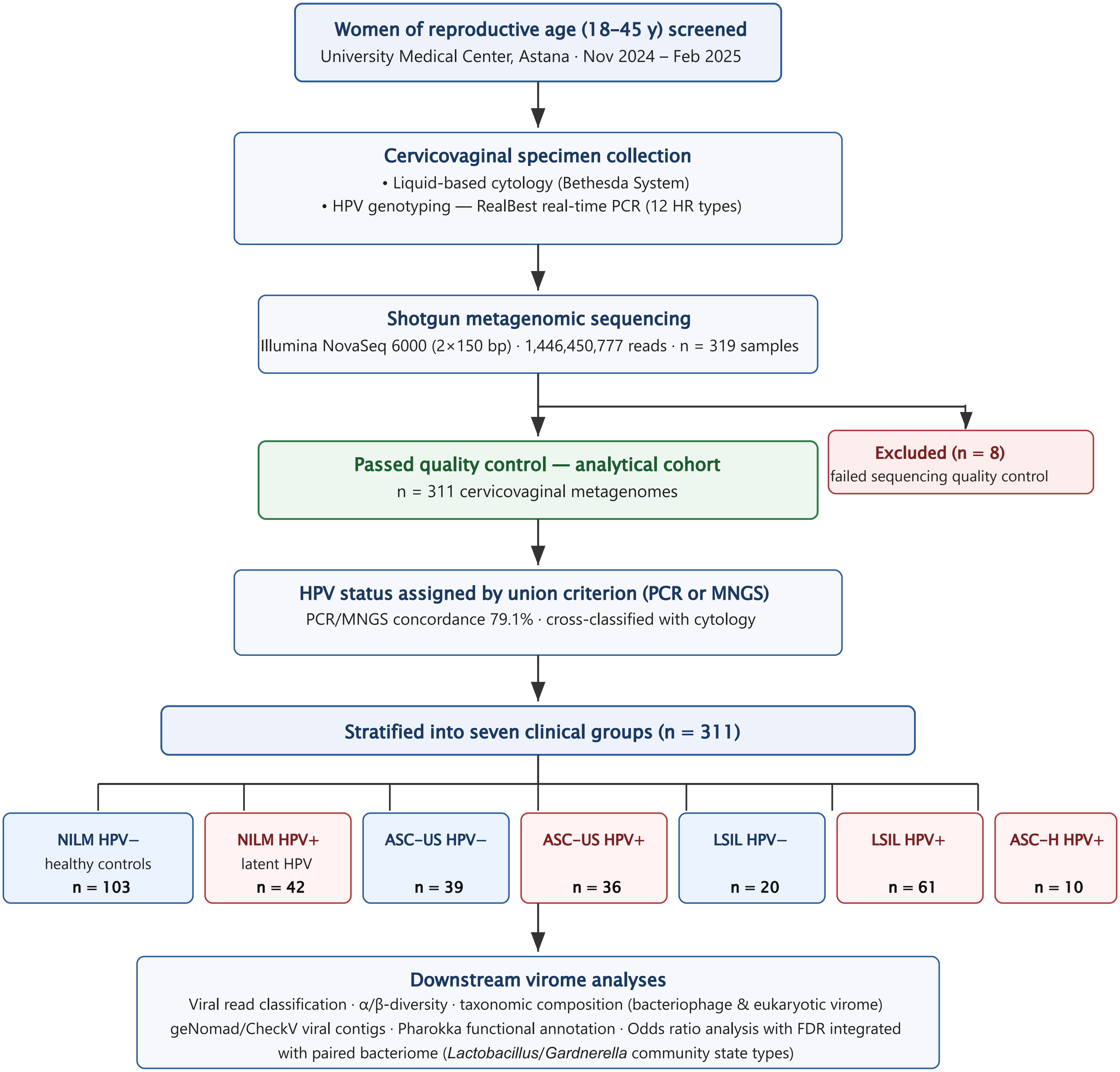

Group colours: blue = HPV-negative strata; red = HPV-positive strata; MNGS, shotgun metagenomic sequencing; NILM, negative for intraepithelial lesion or malignancy; ASC-US, atypical squamous cells of undetermined significance; LSIL, low-grade squamous intraepithelial lesion; ASC-H, atypical squamous cells — cannot exclude HSIL.
