# Supplementary material for: Cervicovaginal Virome Restructuring Associated with HPV Status, Bacterial Dysbiosis, and Cervical Cytological Abnormalities
Source: Viruses. 2026 Sep 19;18(9):1036. doi: 10.3390/v18091036 (PMC13611935; doi:10.3390/v18091036)
Supplement: Supplementary file 1 [file viruses-18-01036-s001.zip › Supplementary_Tables.pdf]

Supplementary Table S1. STROBE Statement - Checklist of items that should be included in reports of cross-sectional studies

Section / Topic	Item	No.	STROBE recommendation	Reported in manuscript (section / location)
Title and abstract				
	Title and abstract	1	(a) Indicate the study's design with a commonly used term in the title or abstract. (b) Provide an informative and balanced summary of what was done and what was found.	Title ("shotgun metagenomic"/cross-sectional cohort); Abstract — structured (Background, Methods, Results, Conclusions), n = 311 across seven clinical groups.
Introduction				
	Background / rationale	2	Explain the scientific background and rationale for the investigation being reported.	Introduction, paragraphs 1–6 (cervicovaginal microbiome–HPV axis; virome knowledge gap).
	Objectives	3	State specific objectives, including any prespecified hypotheses.	Introduction, final paragraph (three-part hypothesis on virome restructuring).
Methods				
	Study design	4	Present key elements of study design early in the paper.	Methods — Study design and participants, paragraph 1 (cross-sectional observational study).
	Setting	5	Describe the setting, locations, and relevant dates, including periods of recruitment, exposure, follow-up, and data collection.	Methods — Study design and participants (University Medical Center, Nazarbayev University, Astana; November 2024 – February 2025).
	Participants	6	(a) Give the eligibility criteria, and the sources and methods of selection of participants.	Methods — Study design and participants (eligibility; seven groups cross-classified by cytology × HPV status, PCR ∪ MNGS).
	Variables	7	Clearly define all outcomes, exposures, predictors, potential confounders, and effect modifiers. Give diagnostic criteria, if applicable.	Methods — HPV genotyping and dual-method verification; Statistical analysis (Cell Status, HPV type distribtution, Age, BMI, Parity, Marital Status, Number of sex partners, community state type (CST))
	Data sources / measurement	8	For each variable of interest, give sources of data and details of methods of assessment (measurement). Describe comparability of assessment methods if there is more than one group.	Methods — Clinical examination and sample collection; HPV genotyping (RealBest real-time PCR, Ct ≤ 35); DNA extraction and shotgun sequencing.
	Bias	9	Describe any efforts to address potential sources of bias.	Methods — Sequencing quality control and contamination assessment (positive/negative extraction controls); negative controls not sequenced → prevalence-based decontamination not applicable (stated as a limitation in Discussion).
	Study size	10	Explain how the study size was arrived at.	Methods — Study design and participants; Results — Study cohort and sequencing output (319 sequenced → 311 after QC; convenience clinical sample).

	Quantitative variables	11	Explain how quantitative variables were handled in the analyses. If applicable, describe which groupings were chosen and why.	Methods — Statistical analysis. Odds ratio analysis with 95% confidence intervals and FDR-BH correction; cytological ordinal grouping NILM→ASC-H).
	Statistical methods	12	(a) Describe all statistical methods, including those used to control for confounding. (b) Methods to examine subgroups and interactions. (c) How missing data were addressed. (d) Sampling strategy. (e) Sensitivity analyses.	Methods — Statistical analysis (Wilcoxon/Kruskal–Wallis; Fisher's exact test with Benjamini–Hochberg FDR; complete-case analysis). Subgroups: Lactobacillus- and CST-stratified analyses.
Results				
	Participants	13	(a) Numbers at each stage. (b) Reasons for non-participation. (c) Consider a flow diagram.	Results — Study cohort and sequencing output (319 → 311; 8 excluded after QC); Supplementary Figure S1 (study flowchart).
	Descriptive data	14	(a) Characteristics of study participants and information on exposures and potential confounders. (b) Number of participants with missing data. (c) Follow-up time (cohort).	Table 1 (age, BMI, reproductive history, HPV status, Marital satus, Number of sexual partners, CST by group); per-group counts or medians are reported; complete-case (no substantial missingness).
	Outcome data	15	Report numbers of outcome events or summary measures.	Results — Sequencing parameters and virome signal characteristics; Figures 1–5; Tables 2–5 (virome composition, diversity, functional/AMG, lysogeny).
	Main results	16	(a) Unadjusted and confounder-adjusted estimates and their precision (e.g., 95% CI). (b) Category boundaries when continuous variables were categorized. (c) Translate relative risk to absolute risk for a meaningful time period.	Results — Cervical virome composition by HPV status (unadjusted) and OR with 95% CI; diagnostic cut-offs in Methods.
	Other analyses	17	Report other analyses done — e.g., analyses of subgroups and interactions, and sensitivity analyses.	Results — biodiversity and dominant-taxa analyses (Figures 1-2); functional/AMG enrichment (Figure 3).
Discussion				
	Key results	18	Summarise key results with reference to study objectives.	Discussion, paragraphs 1–2 (bacteriophage–papillomavirus polarity; Andhravirus shift).
	Limitations	19	Discuss limitations of the study, taking into account sources of potential bias or imprecision. Discuss both direction and magnitude of any potential bias.	Discussion — Limitations (cross-sectional design; unsequenced negative controls; single-center; samlll ASC-H patients number; causal inference constraints).
	Interpretation	20	Give a cautious overall interpretation of results considering objectives, limitations, multiplicity of analyses, results from similar studies, and other relevant evidence.	Discussion, paragraphs 3–7 (description of potential mechanistic model, Figure 5; comparison with prior cervicovaginal microbiome literature).
	Generalisability	21	Discuss the generalisability (external validity) of the study results.	Discussion — Central Asian / Kazakhstani population context and Limitations (single-center generalizability).
Other information				

	Funding	22	Give the source of funding and the role of the funders for the present study and, if applicable, for the original study on which the present article is based.	Funding — Science Committee, Ministry of Science and Higher Education of the Republic of Kazakhstan (Grant No. BR24992853); funder had no role in design, analysis, or reporting.

An Explanation and Elaboration article discusses each checklist item [24]. The STROBE checklist is best used in conjunction with that article. Items marked FLAG indicate points the authors should finalize before submission.

Supplementary Table S2. STORMS checklist. Strengthening The Organization and Reporting of Microbiome Studies: reporting checklist

Section	No.	STORMS element	Item description	Reported in manuscript (section / location)
1. Introduction				
	1.1	Study design / background	Describe the rationale, scope, and primary question; identify the study as a microbiome study.	Introduction, paragraphs 1–6; final paragraph (hypothesis). Title identifies shotgun metagenomic virome study.
	1.2	Hypotheses / objectives	State the specific objectives and any prespecified hypotheses.	Introduction, final paragraph (three-part hypothesis: phage collapse, eukaryotic-virome narrowing, reciprocal dysbiosis amplification).
2. Methods — Study design				
	2.1	Study design and setting	Describe the study design and setting, including location and dates.	Methods — Study design and participants (cross-sectional; UMC, Nazarbayev University, Astana; Nov 2024 – Feb 2025).
	2.2	Participants / eligibility	Eligibility criteria and how participants/samples were selected; groups defined.	Methods — Study design and participants (inclusion/exclusion; seven cytology × HPV groups; PCR ∪ MNGS union criterion).
	2.3	Ethics	State ethics approval, approval number, and that informed consent was obtained.	Methods — Ethics approval and consent to participate (Local Bioethics Committee, University Medical Center, Protocol No. 5, dated 23 May 2024; written informed consent).
	2.4	Sample-size rationale	Describe how sample size was determined (or note convenience sampling).	Results — Study cohort and sequencing output (n = 311 after QC from 319; clinical convenience sample spanning the full cytological spectrum).
3. Methods — Sample collection and lab				
	3.1	Sample collection	Describe specimen type, collection method, and anatomical site.	Methods — Clinical examination and sample collection (cervicovaginal specimens; liquid-based collection).
	3.2	Sample processing / storage	Storage conditions and time from collection to processing.	Methods — Clinical examination and sample collection / DNA extraction (specimens stored at −80 °C until extraction).
	3.3	Nucleic-acid extraction	Describe DNA/RNA extraction method and kit.	Methods — DNA extraction and shotgun metagenomic sequencing (ZymoBIOMICS DNA Miniprep Kit; Qubit quantification).

	3.4	Positive and negative controls	Report use of positive and negative controls in extraction and sequencing.	Methods — DNA extraction (negative extraction control = nuclease-free water; positive control = ZymoBIOMICS Microbial Community Standard, D6300). Negative controls were not sequenced (acknowledged as a limitation).
	3.5	Sequencing method	Describe sequencing platform, read configuration, and target depth.	Methods — DNA extraction and shotgun metagenomic sequencing (Illumina NovaSeq 6000, 2 × 150 bp; 1,446,450,777 reads; median ~45.6 M reads/sample).
	3.6	Analytic sample / flow	Provide a flow diagram or account of samples from collection to analysis.	Supplementary Figure S1 (study flowchart); 319 sequenced → 8 excluded by QC → 311 analyzed.
4. Methods — Bioinformatics				
	4.1	Quality control of reads	Describe read QC, trimming, and host-read removal.	Methods — Bioinformatic pipeline (fastp v0.23.4; host depletion against the human reference with Bowtie2).
	4.2	Sequence analysis / assembly	Describe assembly and viral-sequence identification tools and versions.	Methods — Bioinformatic pipeline (MEGAHIT assembly; geNomad mobile-element/virus identification; CheckV completeness/quality).
	4.3	Taxonomic classification	Describe taxonomic profiling tools, versions, and reference databases.	Methods — Bioinformatic pipeline (Kraken2 v2.1.3 PlusPF database (k2_pluspf, build 20251015); Pharokka functional annotation of viral contigs).
	4.4	Contaminant identification	Describe how contaminants were identified/removed (e.g., decontam).	Methods — Sequencing quality control and contamination assessment. Prevalence-based decontamination (decontam) NOT applied because negative controls were not sequenced; stated transparently as a limitation.
	4.5	Data transformation / normalization	Describe normalization or transformation of feature tables.	Methods — Statistical analysis (Odds ratio analysis with 95% confidence intervals and FDR-BH correction).
5. Methods — Statistics				
	5.1	Statistical analysis overview	Describe software (with versions) and the overall analytic strategy.	Methods — Statistical analysis (Python v3.9.16; R v4.2.2; analytic workflow for composition, diversity, and differential prevalence with odds ratios with confidence intervas with FDR-BH procedure).
	5.2	Diversity analyses	Describe alpha- and beta-diversity metrics and statistical tests.	Methods — Statistical analysis (Observed species, Faith's PD; unweighted UniFrac; ANOSIM, 999 permutations).
	5.3	Differential abundance	Describe differential-abundance methods and multiple-testing correction.	Methods — Statistical analysis (Fisher's exact test with Benjamini–Hochberg FDR).
	5.4	Confounders and covariates	List covariates/confounders	No covariates were analyzed
6. Results and Discussion				
	6.1	Participant characteristics	Report participant/sample characteristics by group.	Table 1 (age, BMI, reproductive history, HPV status, Marital satus, Number of sexual partners, CST by group).

	6.2	Sequencing output	Report sequencing yield and quality metrics.	Results — Study cohort and sequencing output / Sequencing parameters (total 1,446,450,777 reads; viral read fraction; median depth and range).
	6.3	Key findings	Report primary microbiome findings with effect sizes and uncertainty.	Results — all subsections; Figures 1–4; Tables 1–5 (virome composition, diversity, functional/AMG landscape, lysogeny–lysis switching).
	6.4	Data and code availability	State where raw data, processed data, and analysis code are available.	Methods — Availability of data and materials: raw metagenomic data deposited at NCBI BioProject PRJNA1337009.
	6.5	Reporting checklist	State that a reporting checklist (STORMS) is provided.	Supplementary Table S2 (this STORMS checklist); Supplementary Table S1 (STROBE).

[25]Reporting guidelines for human microbiome research: the STORMS checklist. Nat Med 2021;27:1885–92. Items marked FLAG indicate points the authors should finalize before submission to Microbiome.

Supplementary Table S3. Classification of human papillomavirus (HPV) genotypes into Alphapapillomavirus species-level groups (α-groups) according to NCBI Taxonomy and the ICTV Papillomaviridae framework.

α-group (species)	HPV genotypes (members)	Risk class	Notes / relevance to this study
Alphapapillomavirus 9 (α-9)	HPV16, 31, 33, 35, 52, 58, 67	High-risk	Dominant oncogenic clade; accounts for ~75% of invasive cervical cancers. Progressive enrichment toward this clade with lesion severity in this cohort (59.7% of HPV+ cases).
Alphapapillomavirus 7 (α-7)	HPV18, 39, 45, 59, 68, 70	High-risk	Accounts for ~15% of cervical cancers; disproportionately associated with adenocarcinoma. 21.0% in this cohort.
Alphapapillomavirus 5 (α-5)	HPV26, 51, 69, 82	High/possible-risk	HPV51 high-risk (IARC Group 1); HPV26, 82 possibly carcinogenic. 12.6% in this cohort.
Alphapapillomavirus 6 (α-6)	HPV30, 53, 56, 66	High/possible-risk	HPV56 high-risk (Group 1); HPV53, 66 possibly carcinogenic (Group 2B). 17.6% in this cohort.
Alphapapillomavirus 3 (α-3)	HPV61, 62, 72, 81, 83, 84, 89	Low-risk	Predominantly low-risk mucosal types. 17.6% in this cohort.
Alphapapillomavirus 10 (α-10)	HPV6, 11, 44	Low-risk	Classic low-risk types associated with genital warts. 12.6% in this cohort.
Alphapapillomavirus 11 (α-11)	HPV34, 73	Possible-risk	HPV73 possibly carcinogenic (Group 2B). HPV34 resolved by MNGS only (Figure 1C); not in the targeted PCR panel.
Alphapapillomavirus 13 (α-13)	HPV54	Low-risk	Low-risk mucosal type. Resolved by MNGS only (Figure 1C); not in the targeted PCR panel.

Notes. α-group assignments follow the NCBI Taxonomy browser (Alphapapillomavirus) and the ICTV Papillomaviridae classification, consistent with Chen et al. [45]and Chen et al [46]. Risk classification follows the IARC monograph: Group 1 (carcinogenic): HPV16, 18, 31, 33, 35, 39, 45, 51, 52, 56, 58, 59; Group 2A (probably carcinogenic): HPV68; Group 2B (possibly carcinogenic): HPV26, 53, 66, 67, 70, 73, 82. The targeted PCR panel used in this study (RealBest) detects HPV16, 18, 31, 33, 35, 39, 45, 51, 52, 56, 58, 59, spanning α-5, α-6, α-7 and α-9; additional genotypes (e.g., α-3, α-10, α-11 and α-13 members) were resolved by MNGS only. Several cutaneous Beta- and Gammapapillomavirus types (HPV7, 90, 121, 127, 134, 144, 156) were also detected by MNGS at low abundance and are shown in Figure 1C; these are not Alphapapillomavirus mucosal types and are not assigned an α-group here. Genotypes are listed by their established species-group membership; not all members occur in the present cohort.
